# Rapid Discriminative Identification of the Two Predominant Echinococcus Species from Canine Fecal Samples in the Tibetan Region of China by Loop-Mediated Isothermal Amplification–Lateral Flow Dipstick Assay

**DOI:** 10.3390/tropicalmed9060136

**Published:** 2024-06-20

**Authors:** Xinyue Lv, Jiajia Ai, Xiaojin Mo, Haojie Ding, Sofia Litchev, Entung Lu, Youhong Weng, Qing He, Quzhen Gongsang, Shijie Yang, Xiumin Ma, Jingzhong Li, Huasheng Pang, Shaohong Lu, Qingming Kong

**Affiliations:** 1 Key Laboratory of Bio-Tech Vaccine of Zhejiang Province, Engineering Research Center of Novel Vaccine of Zhejiang Province, School of Basic Medicine and Forensics, Hangzhou Medical College, Hangzhou 310013, China; lvxinyue215@163.com (X.L.);; 2 Tibet Center for Disease Control and Prevention, NHC Key Laboratory of Echinococcosis Prevention and Control, Lhasa 850000, China; vencyjia@163.com (J.A.);; 3 National Institute of Parasitic Diseases, Chinese Center for Disease Control and Prevention, Chinese Center for Tropical Diseases Research, National Key Laboratory of Intelligent Tracking and Forecasting for Infectious Diseases, Key Laboratory on Parasite and Vector Biology, Ministry of Health, Shanghai 200025, China; 4 Department of Chemistry & Biochemistry, University of California, Los Angeles, CA 90095, USA; 5 Santa Monica College, Los Angeles, CA 90405, USA; 6 State Key Laboratory of Pathogenesis, Prevention and Treatment of High Incidence Diseases in Central Asia, Clinical Laboratory Center, Tumor Hospital Affiliated to Xinjiang Medical University, Urumqi 830000, China; 7 Key Laboratory of Biomarkers and In Vitro Diagnosis Translation of Zhejiang Province, School of Laboratory Medicine and Bioengineering, Hangzhou Medical College, Hangzhou 310013, China

**Keywords:** echinococcosis, LAMP, repetitive sequences, dry fluorescence immunoassay analyzer, lateral flow dipstick, prevalence

## Abstract

Echinococcosis poses a significant concern in the fields of public health and veterinary care as it can be transmitted between animals and humans. The primary endemic subtypes are cystic echinococcosis (CE) and alveolar echinococcosis (AE), which result from infestation by *Echinococcus granulosus* and *Echinococcus multilocularis*, respectively. A prominent epidemic of echinococcosis greatly affects the Tibet Autonomous Region (TAR) in China. A new technique called the loop-mediated isothermal amplification–lateral flow dipstick (LAMP-LFD) test is introduced in this research to differentiate between *E. granulosus* and *E. multilocularis* using their repetitive genetic sequences. The test is characterized by its portable nature, simple operation, quick result production, high sensitivity, and low susceptibility to aerosol contamination. The LAMP-LFD method demonstrated an exceptional minimal detection limit, reaching levels as low as approximately 1 fg/μL (femtogram per microliter) of genomic DNA. The assay’s specificity was assessed, and no cross-reactivity was seen. A total of 982 dog fecal samples were collected from 54 counties in the TAR region between July 2021 and June 2022. The established method underwent validation using a commercially available ELISA kit. The agreement rate between the LAMP-LFD and ELISA methods was 97.25%, with a sensitivity of 96.05% and a specificity of 97.35%. The assay described in this study improves specificity by using a double-labeled probe, and it reduces the risk of false-positive results caused by aerosol contamination through the use of a sealed device. This makes it a suitable choice for quickly and accurately identifying the two main types of *Echinococcus* in field settings.

## 1. Introduction

Echinococcosis, or hydatid disease, is a prevalent parasitic infection that affects both humans and animals globally. The World Health Organisation lists it as one of the 17 neglected tropical diseases. It poses a significant risk to human health and safety and also places a substantial financial strain on animal husbandry. The two most prevalent species of *Echinococcus* tapeworms that parasitize people are *Echinococcus granulosus* and *Echinococcus multilocularis*, which give rise to cystic echinococcosis (CE) and alveolar echinococcosis (AE), respectively. As per the epidemiological reference group on foodborne disease burden calculation by the World Health Organisation (WHO), approximately 19,300 fatalities and 871,000 disability-adjusted life years (DALYs) are lost worldwide each year owing to CE [1]. The yearly expenses related to CE, encompassing medical care and livestock losses, amount to almost USD 3 billion [2]. The adverse effects of AE are notably consequential, and the disease is commonly referred to as “insect cancer” or “second cancer.” Analysis of clinical cases reveals that more than 95% of adverse events stem from the liver. If patients do not receive therapy promptly, their mortality rate during a span of 10–15 years exceeds 90% [3]. China has a high incidence of echinococcosis, particularly in the large agricultural and pastoral regions of Western and Northern China. The prevalence of echinococcosis was 1.66%, with an estimated 50,000 cases in 74 counties within the Tibet Autonomous Region (TAR) [4]. Among animals, the prevalence was 7.30% in dogs and 13.21% in domestic animals (cattle and sheep) [5]. The infections are highly correlated with the prevalence of human encopresis [6].

Dogs serve as the definitive host for *Echinococcus*, with the adult-stage parasite residing in the small intestine of dogs. Canine echinococcosis is prevalent in rural regions, as dogs serve as companions to herdsmen and protectors of their livestock [7]. Several methods exist for detecting echinococcosis around the world, the most common one being Arecoline [8]. However, this process is highly time-consuming, necessitates skilled staff, and carries the potential danger of *Echinococcus* infection. The dog fecal antigen approach is frequently employed to detect canine *Echinococcus* infection, serving as a prevalent screening technique for parasites. Researchers greatly value the technology’s maturity and exceptional sensitivity [9]. However, this method sometimes poses difficulties in achieving the required results of detection, and it fails to distinguish between infections produced by *E. granulosus* and *E. multilocularis*. To enhance the precision of diagnosing *Echinococcus* infection and determine the origin of infection in dog feces, several methods were utilized. These methods include polymerase chain reaction (PCR), nested PCR, multiplex PCR, and real-time quantitative PCR [10,11,12,13,14]. These techniques based on PCR were employed to directly identify the DNA of the *Echinococcus* parasite in canine fecal samples. However, these methods require complex and expensive equipment and are not convenient for grassroots hospitals and on-site detection.

Loop-mediated isothermal amplification (LAMP) assays have been developed for the detection of *E. multilocularis* [15] and *E. granulosus* [16] in fecal samples. These assays are known for their simple amplification conditions, easy operation, and high specificity [17]. Integrating lateral flow dipsticks (LFDs) has enhanced the sensitivity and specificity of LAMP product identification, resulting in increased performance. Within these optimization initiatives, the LAMP-LFD method has been employed to identify a range of parasites, including *Toxoplasma gondii* [18], *Babesia bovis* and *Babesia bigemina* [19], and the African trypanosome [20]. In traditional LAMP-LFD methods, researchers open the reaction tube to transfer the reaction product to the LFD once LAMP is complete. But because the LAMP process is very sensitive and easily contaminated, any aerosol contaminants made in previous LAMP reactions can be used as templates for amplification, which can lead to false-positive results.

The current study aimed to develop an integrated system capable of efficiently, precisely, easily, and fully self-sealing to visualize nucleic acid amplification products on LFD strips, eliminating the need to open the reaction tube. This device enables easy attainment of the result using a typical laboratory water bath. The screening results revealed that two sets of primers, one demonstrating high sensitivity to both *E. granulosus* and *E. multilocularis* and the other specifically sensitive to *E. granulosus*, effectively discriminated between the two species. This significant finding holds promise for advancing echinococcosis prevention and control efforts. The primary objective of the present investigation was to evaluate the effectiveness of the LAMP-LFD detection method by detecting the DNA of both *E. granulosus* and *E. multilocularis* in canine fecal samples obtained from seven cities in the TAR.

## 2. Materials and Methods

### 2.1. Tibet Autonomous Region Parasites and Samples

The Lanzhou Veterinary Research Institute of the Chinese Academy of Agricultural Sciences kindly provided the positive animal tissue samples of *E. multilocularis* and *E. granulosus*. A total of 982 canine fecal samples were collected from 54 areas exhibiting a high frequency of the disease among seven cities in the TAR.

### 2.2. Assessment of ELISA

ELISA analysis was performed on the canine fecal samples, adhering to the instructions supplied by the commercially available reagent kit specifically developed for detecting the antigen of *Echinococcus*. The kit was supplied by Shenzhen Combined Biotech Co., Ltd. (Shenzhen, China) and utilized a double-antibody sandwich ELISA assay. One gram of feces was combined with sample buffer, and 100 µL of the resulting fecal slurry was subjected to an antigen test. If the sample contains a soluble antigen, it can bind to the antibody components of the assays. For each antigen test, a positive sample, a negative sample, and three essential control samples were utilized. The microplates were analyzed using a spectrophotometer set to a wavelength of 450 nm. The critical mean was exceeded to determine a positive result based on the optical density (OD). If the positive control had a value over 0.3, the negative control had an OD value lower than the critical mean, and the critical mean itself was higher than 0.15, the plate test was deemed valid. All dog fecal samples were subsequently sent to the Tibet Autonomous Region Centre for Disease Control and Prevention for further confirmation.

### 2.3. DNA Extraction

Genomic DNA was extracted from the positive animal tissues using a TIANamp Genomic DNA Extraction Kit (TIANGEN, Beijing, China), according to the manufacturer’s instructions. The Fast DNA™ SPIN Kit for Soil (MP, Santa Ana, CA, USA) was used to extract DNA from canine fecal samples.

To enhance the yield and detection rate of A, up to 500 mg of canine feces sample, along with 978 μL of Sodium Phosphate Buffer and 122 μL of MT Buffer, were placed into the Lysing Matrix E tube. It was left in a 4 °C freezer overnight for thorough lysis. Then, it was eluted with 30 μL of elution buffer. The DNA concentration was assessed using a NanoDrop spectrometer (Thermo Fisher Scientific, Waltham, MA, USA) and subsequently kept at −20 °C until further use.

### 2.4. Design of Primers and Probe

**Figure 1:**
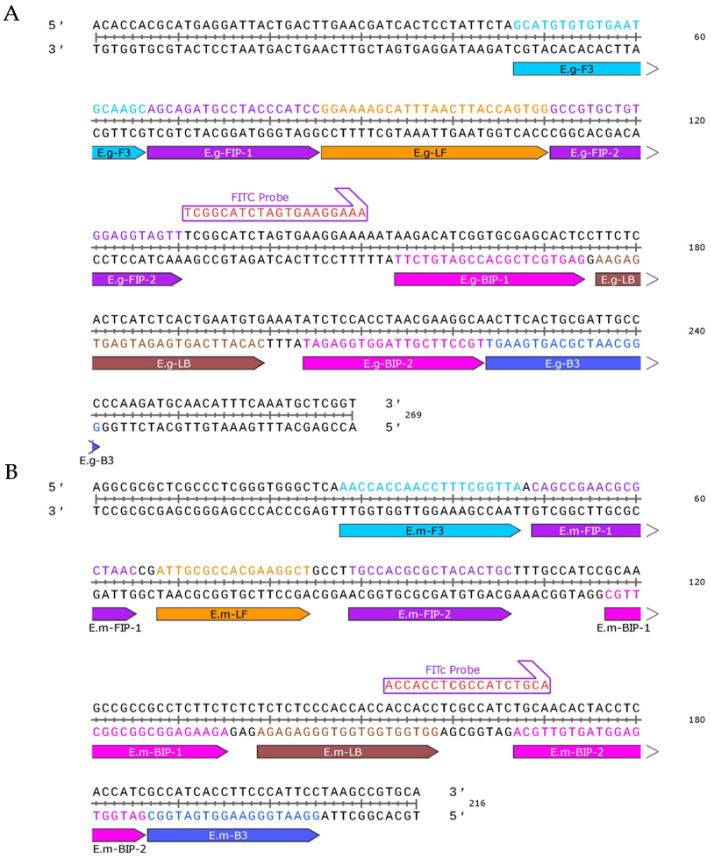
The nucleotide sequence of *Echinococcus granulosus*-RRS (**A**) and *Echinococcus multilocularis*-RRS (**B**) shows the set of primers. The sequences marked with sky blue, purple, orange, purple, pink, brown, pink, blue, and red represent primers F3, FIP-1, LF, FIP-2, BIP-1, LB, BIP-2, B3, and FITC-Probe, respectively. The forward inner primer (FIP) was labeled with biotin at the 5′ end, with amplification in the 5′→3′ direction.

Prior research has shown that using 130 to 200 bp DNA with LAMP technology yields optimal detection outcomes [17]. The online LAMP primer designing software Primer Explorer V5 (http://primerexplorer.jp/e/v5_manual/index.html (accessed on 10 September 2022)) was used to make two sets of specific oligonucleotide primers that targeted the gene bank: KR347168.1 E.g repeat region sequence and the gene bank: AF492849.1 E.m repeat region sequence. The 5′ end of the forward inner primer FIP was labeled with biotin, and the probe was labeled with FITC. This was designed between primers B1c and B2 for molecular hybridization detection of FITC-biotinylated LAMP products (Figure 1). Table 1 lists the designed primer sequences.

### 2.5. Construction of Positive Plasmids

The genomic DNA of *E. granulosus* and *E. multilocularis* was separately amplified using the required primers F3 and B3 by PCR. The resulting products were then analyzed using 1% agarose gel electrophoresis. The specific and strong bands that matched the expected size were cut out of the agarose gel using UV light. The desired gene was then isolated using the UE DNA Gel Extraction Kit (UE, Suzhou, China) and subsequently combined with the pET-19T vector (Takara, Beijing, China) through ligation. The ligation product was introduced to the Fast-T1 competent cells (Vazyme, Nanjing, China) and evenly distributed over LB solid medium supplemented with ampicillin. After incubating the cells at 37 °C overnight. PCR amplification of bacterial liquid was performed on five individual colonies, and the positive samples were subsequently selected for expansion and plasmid extraction. Sequencing and analysis of the obtained recombinant plasmids successfully identified correctly generated positive plasmids for both *E. granulosus* and *E. multilocularis*.

### 2.6. The Real-Time LAMP Reaction System

The LAMP reaction system was established by taking into account prior studies [17], and it was further optimized by considering the specific characteristics of *E. granulosus* genes. The optimized reaction system had a total 25 μL reaction mixture containing the specified amounts genomic DNA, 20 mM Tris-HCl (pH 8.8), 50 mM KCl, 10 mM (NH_4_)_2_SO_4_, 2 mM MgSO_4_, 0.1% Tween-20, 5 pmol each of F3 and B3, 40 pmol each of BIP and biotin-FIP, 40 pmol LF and LB, 1 μL of Bst 2.0 WarmStartÒ DNA polymerase (New England Biolabs, Beijing, China), 1 mM dNTPs (New England Biolabs, Beijing, China), and 0.5 μL fluorescent dye enzyme (New England Biolabs, Beijing, China) was used for real-time LAMP, with amplification performed on a CFX96 Touch™ Real-Time PCR Detection System (Bio-Rad, Hercules, CA, USA). The acquisition of the fluorescent signal determined the amplification products.

### 2.7. LAMP–Lateral-Flow Dipstick (LAMP-LFD)

**Figure 2:**
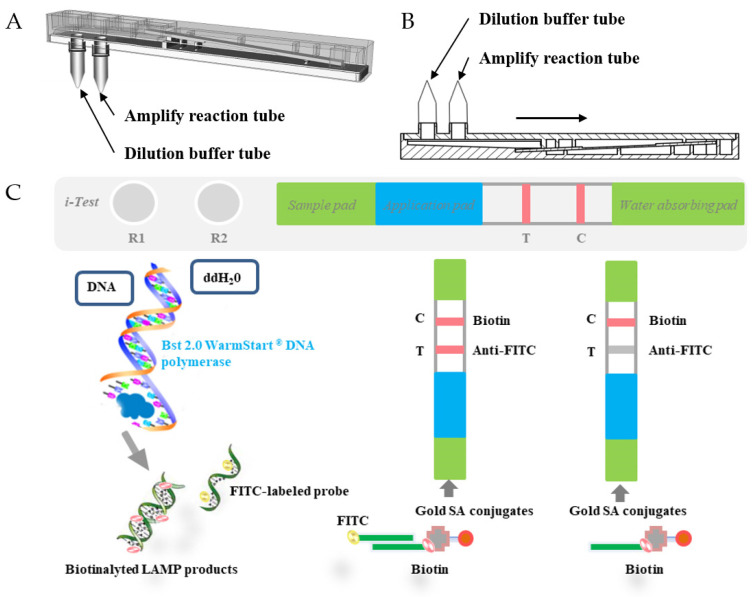
The LAMP-LFD device and principle. (**A**) Schematic representation of the LAMP-LFD model, (**B**) side view of LAMP-LFD equipment, and (**C**) illustration depicting the operational concept of the LAMP-LFD method.

To detect the amplification products of the LAMP reaction, a device was designed that is well sealed, easy to carry, and has visualized results. The device combines the LAMP reaction with lateral flow strip paper (Figure 2). Two slots at the bottom of the device allow for the installation of nucleic acid amplification reaction tubes and dilution liquid tubes. Next to the reaction part of the device is a visual result reading box that concaves inward for observing the nucleic acid amplification reaction. Inside the reading box is a bracket that can fix the nucleic acid detection strip paper horizontally and vertically. The LFD reaction device includes a nucleic acid reaction decompression buffer chamber, a latex pad that blocks steam generated during the reaction heating process, and a plastic grooved pedestal for positioning the nucleic acid detection strip paper. The lateral flow dipstick consists of sample and application pads, test and control lines, and a water-absorbing pad, all set on the plastic grooved pedestal. Streptavidin (SA) conjugate, anti-FITC mouse monoclonal antibody, and biotin cover the application pad, test line, and control line, respectively. Before starting the test, the user only needs to mix the test sample with the LAMP reaction mixture and add it to the reaction tube, then connect the reaction tube and dilution tube to the corresponding slots, and finally put them in a 65 °C constant temperature water bath for 1 h to complete the LAMP reaction. After the required product amplification is complete, the device is closed, and the FITC-biotinylated nucleic acid product is mixed with the nucleic acid dilution buffer. The mixture flows to the lateral flow strip, binds with the Gold-SA conjugate, and forms a triple-labeled complex when passing through the application pad. It then moves up the strip. The fixed anti-FITC antibody (test line) captures it. The fixed biotin captures the double complex formed by the biotin-labeled FIP primer and the Gold-SA conjugate without FITC (control line). If both the control line and the test line are visible in the visualized result reading box, the result is positive. If only the control line is visible, the result is negative. The LAMP-LFD gadget was utilized to identify the presence of *E. granulosus* and *E. multilocularis* in animal fecal samples that were previously screened using commercial ELISA kits.

### 2.8. Assessment of the Specificity and Sensitivity of the LAMP-LFD Method

The genomic DNA of *E. multilocularis*, *Sparganum manson*, *Trypanosoma evansi*, and *Schistosoma japonicum* were utilized as templates in accordance with the reaction system and process of the LAMP and LAMP-LFD methods indicated earlier. The amplification of *E. granulosus* and *E. multilocularis* was carried out using specialized primers developed for the LAMP and LAMP-LFD methods, respectively.

The sensitivity of the LAMP-LFD method was assessed by testing a succession of diluted positive plasmids, with each dilution being ten times less concentrated than the previous one. The dilution range for positive plasmids of *E. granulosus* ranged from 1.6 pg to 0.0016 fg, while the dilution range for positive plasmids of *E. multilocularis* ranged from 140 pg to 0.14 fg. Non-nucleic acid enzyme water was employed as a negative control. Each concentration underwent three trials for both the LAMP and LAMP-LFD techniques.

## 3. Results

### 3.1. Specificity of the Established LAMP-LFD Method

**Figure 3:**
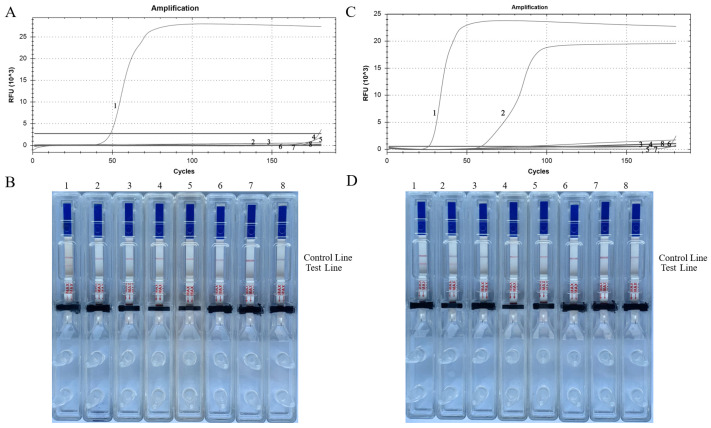
Specificity verification of LAMP *E*. *granulosus* (**A**,**B**) and *E. multilocularis* (**C**,**D**) primers. (**A**,**C**) Curves for real-time LAMP. (**B**,**D**) Visual inspection of LAMP-LFD tests. (1) *E*. *granulosus*; (2) *E. multilocularis*; (3) mouse tapeworm; (4) Sparganum isolate from Henan; (5) Sparganum Guangdong isolate; (6) *Schistosoma japonicum*; (7) *Angiostrongylus cantonensis*; (8) negative control.

Genomic DNA samples were used from *E. granulosus*, *E. multilocularis*, *S. manson*, *T. evansi*, and *S*. *japonicum* to test the specificity of the LAMP and LAMP-LFD methods. When using the primers specifically designed for *E. multilocularis*, both *E. granulosus* and *E. multilocularis* demonstrated amplification. Figure 3A,B shows that there was no cross-reaction with other parasites. Conversely, when utilizing the primers designed for *E. granulosus*, only *E. granulosus* exhibited amplification using these two methods. Figure 3C,D depicts the absence of signals from other DNA templates. Therefore, in practice, it is recommended to use *E. multilocularis* primers initially to determine whether the sample is *Echinococcus*. If the result is positive, *E. granulosus* can be used for species identification. The results show that the tried-and-true methods are not only very good at finding *Echinococcus*, but they can also tell the difference between *E. granulosus* and *E. multilocularis*.

### 3.2. Sensitivity of the Proven LAMP-LFD Method

**Figure 4:**
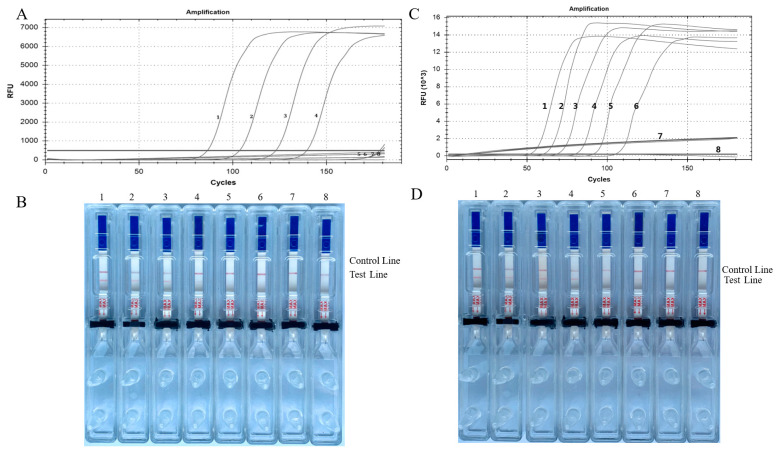
Sensitivity of *Echinococcus granulosus* (**A**,**B**) and *Echinococcus multilocularis* (**C**,**D**) LAMP primers. (**A**,**C**) Curves for real-time LAMP and (**B**) visual inspection of LAMP-LFD, (1) 1.6 pg/μL, (2) 160 fg/μL, (3) 16 fg/μL, (4) 1.6 fg/μL, (5) 0.16 fg/μL, (6) 0.016 fg/μL, (7) 0.0016 fg/μL, (8) negative control. (**D**) Visual inspection of LAMP-LFD, (1) 140 pg/μL, (2) 14 pg/μL, (3) 1.4 pg/μL, (4) 140 fg/μL, (5) 14 fg/μL, (6) 1.4 fg/μL, (7) 0.14 fg/μL, (8) negative control.

A sensitivity analysis was performed on the LAMP-LFD method utilizing genomic DNA samples at varying doses. The plasmid template containing *E. granulosus* was diluted to a concentration of 0.0016 fg, which is ten times greater than 1.6 pg. Real-time LAMP and LAMP-LFD tests were then performed. The signal curve for the real-time LAMP method showed that 1.6 fg of plasmid DNA was the lowest amount that could be detected after an hour. The LAMP-LFD method showed similar findings, as illustrated in Figure 4A,B. The plasmid template containing *E. multilocularis* was diluted to a concentration of 0.14 fg, which is ten times greater than 140 pg. Real-time LAMP and LAMP-LFD tests were then performed. Real-time LAMP demonstrated a minimum detectable amount of 1.4 fg of plasmid DNA at one hour, and comparable outcomes were achieved using the LAMP-LFD method (Figure 4C,D).

### 3.3. Dog Fecal Sample Test

The LAMP-LFD approach detected *Echinococcus* DNA in canine fecal samples at a positive rate of 9.88% (97/982), surpassing the positive rate of identifying canine fecal antigen using the ELISA method, which was 7.74% (76/982) (Table 2). We conducted two repeat experiments on the field samples, and the results were consistent. The LAMP-LFD method demonstrated a sensitivity of 96.05% and a specificity of 97.35% when compared to the ELISA method. The overall agreement rate was 97.25%, and the Kappa value was 0.829, indicating a high level of consistency between the Echinococcus DNA detection results obtained using our LAMP-LFD method and the commercially available ELISA fecal antigen method (Table 3).

## 4. Discussion

The TAR is one of the areas in China with the most severe prevalence of echinococcosis. Given the severe repercussions and worldwide impact of echinococcosis, as well as the absence of efficient medications, it is crucial to devise prompt, accurate, and economical diagnostic techniques and on-site testing. This is necessary for the timely identification, prevention, management, and treatment of echinococcosis. Due to its high sensitivity and specificity, LAMP has been widely used in the diagnosis of echinococcosis. The application of the LAMP method was initially employed for the detection of *E. granulosus* infection. The method involves the use of four LAMP reaction primers based on a part of the *E. granulosus* mitochondrial 12S rRNA sequence that stays the same. Experimental validation demonstrated that the primers exhibited no cross-reactivity with *Taenia saginata* and displayed a distinct level of specificity. Experimentation with various concentrations of *E. granulosus* eggs simultaneously revealed the identification of DNA samples containing a single egg, demonstrating the exceptional sensitivity of the method [21]. Supplementary primers, known as loop primers, expedited the LAMP reaction by reducing the reaction time to less than 50% of the traditional LAMP approach. The overall analysis duration, encompassing detection, is less than one hour, which is beneficial for genetic analysis, including clinical laboratory genetic diagnosis. Using this information, two sets of primers were designed to specifically target the repetitive sequences of *Echinococcus*. Furthermore, the inclusion of loop primers with the internal and external primers improved the efficiency, sensitivity, and specificity of amplification for detecting both *E. granulosus* and *E. multilocularis* [22]. The LAMP reaction system was improved by determining the ideal reaction temperature, reaction time, and concentration of MgSO_4_, using reference [23] as a basis. Observing LAMP products usually involves gel electrophoresis, magnesium pyrophosphate turbidity measurement, or fluorescent dye labeling. However, these methods are costly, require specialized equipment, and can be inconvenient. The LAMP-LFD method uses an expedited and more straightforward LFD technique for ascertaining the outcomes of the reaction. Specifically, the LAMP-LFD method is designed for amplification products generated by molecular probe hybridization techniques, as well as for biotin and fluorescein-labeled LAMP products that are used in a double sandwich configuration. The combination of LAMP with LFD was used to identify hepatopancreatic parvovirus in prawns. The study showed that the LAMP-LFD method is a remarkably sensitive, secure, and expeditious technique [24].

In this study, we applied LAMP and LFD methods for product detection. Specifically, we used biotinylated internal primers and FITC-labeled probes to generate biotinylated and FITC-labeled LAMP products with stem-loop structures at the end. Afterwards, we captured and displayed the results when the product flowed through the anti-FITC antibody region of the lateral flow strip. This method is more specific and sensitive than measuring magnesium pyrophosphate turbidity and using fluorescent dyes. Our results show that the LAMP-LFD method can detect positive plasmids of *Echinococcus* at concentrations as low as 1.6 fg and 1.4 fg within one hour, and there was no cross-reaction with the genomic DNA samples of *S. Mansoni*, *T. evansi*, or *S. japonicum*. During the specificity testing, we found that the primers for *E. multilocularis* can detect both *E. granulosus* and *E. multilocularis*, while the primers for *E. granulosus* can only detect *E. granulosus*. Therefore, our LAMP-LFD method can not only detect *Echinococcus* but also differentiate between *E. granulosus* and *E. granulosus*. In light of the aforementioned considerations, our sample detection process was divided into two distinct stages. The initial step involved testing all samples using the primers for *E. multilocularis.* The screening process yielded positive results for both *E. multilocularis* and *E. granulosus*. In step two, the primers for *E. granulosus* were employed to test the previously identified positive samples from step one. The experimental results indicate a positive result for *E. granulosus*, with the remaining sample identified as *E. multilocularis*. Consequently, the two aforementioned steps enable our LAMP-LFD method to not only detect Echinococcus but also differentiate between *E. multilocularis* and *E. granulosus*.

The LFD antigen detection approach was utilized to assess patients with cystic echinococcosis and revealed a diagnostic sensitivity of 77.14% [22], surpassing the findings of other investigations. Previous studies have also reported a diagnostic specificity of 82.35%, confirming our findings. The type and purity of the antigen used influence the effectiveness of immunodiagnostic testing for cystic echinococcosis [25]. The presence of antigen–antibody immune complexes can also reduce the sensitivity of antigen detection tests for cystic echinococcosis [24]. A LAMP technique was devised to identify *E. granulosus* with a sensitivity of 100 fg per 200 μL of distilled water [26]. Another LAMP technique was developed to detect canine tapeworm with a sensitivity of 1 pg [27]. The LAMP-LFD method we created to detect *E. multilocularis* and *E. granulosus* has a detection sensitivity of 1.4 fg and 1.6 fg.

In addition, aerosol contamination leading to false positives is an inevitable problem in molecular detection methods due to their high sensitivity. The high amplification efficiency of LAMP can achieve 10^9^- to 10^10^-fold nucleic acid amplification in 15~30 min, greatly increasing the risk of aerosol generation. Therefore, minimizing aerosol contamination during LAMP detection remains a top priority in our research. For this reason, we designed a sealed integrated device, and the operator only needs to place the reaction device in a 65 °C water bath. The LAMP-LFD device automatically dilutes and mixes the liquid in the nucleic acid reaction tube and dilution tube in the reaction chamber of the device by inverting once the reaction is completed. The side of the paper strip detects the flow of the nucleic acid mixture, and the visualization window displays the result. The device performs the entire test process, ensuring a closed environment and minimizing the chance of aerosol contamination. The only equipment required is a water bath, making this LAMP reaction a simple, cost-effective, and minimally equipped laboratory and field method. This study designed the LAMP-LFD device to be applicable in various settings. First, this LAMP-LFD detection can detect *Echinococcus* in dog fecal samples and can be extended to a wide range of warm-blooded animals, food samples, etc. This device can perform isothermal amplification of nucleic acids, including the newest RPA isothermal amplification technology. This can quickly find assay products and keep amplification product diffusion contamination to a minimum by switching out certain primers in the LAMP reaction.

In this study, a total of 982 fecal samples were collected from seven different areas in the TAR. The fecal samples were first tested using the ELISA sandwich method in the local cities and then sent to the TAR Centre for Disease Control and Prevention for confirmation. The optimized LAMP-LFD method was used to detect these samples after collection, with a total positive rate of 9.88%. The positive rate using the ELISA method was 6.92%. We performed two repeat tests on the field samples, and the results were consistent, confirming the method’s reliability. The overall agreement rate between the LAMP-LFD method and the ELISA method was 97.25%, with a Kappa value of 0.829. This indicates that the LAMP-LFD nucleic acid detection results were consistent with those obtained using a commercially available ELISA antigen. The above indicators reflect the good authenticity and reliability of the LAMP-LFD detection method. The positive rate of the LAMP-LFD method was higher than that of the ELISA method because the LAMP-LFD method has a higher sensitivity at the molecular level and high amplification efficiency, while the ELISA method is the most sensitive at the protein level. However, it cannot detect samples with very low concentrations or samples with strong interfering factors, and the fecal samples collected in the field usually contain more impurities. Repeated freeze–thawing can also affect the sensitivity of the samples. The LAMP-LFD method we designed with a closed device for the entire process not only minimizes the risk of aerosol pollution but also allows for simple, convenient, and efficient on-site detection.

## 5. Conclusions

Our study developed a closed detection method based on LAMP and LFD for detecting *Echinococcus*. The results indicate that this method is a straightforward and portable molecular diagnostic tool that significantly reduces false positives caused by aerosol pollution while maintaining high detection sensitivity. Additionally, it can specifically differentiate between *E. granulosus* and *E. multilocularis*. Therefore, primary health care institutions, such as township hospitals, can highly benefit from this method, as it is expected to assist in on-site epidemiological investigations of *Echinococcus*.

## Figures and Tables

**Table 1:** LAMP primer sequences for *Echinococcus granulosus* and *Echinococcus multilocularis*.

Primer	Size (bp)	Sequence (5′→3′)
*Echinococcus granulosus*		
Eg-F3	20	GCATGTGTGTGAATGCAAGC
Eg-B3	18	GGGCAATCGCAGTGAAGT
Eg-FIP	39	AACTACCTCCACAGCACGGCAGCAGATGCCTACCCATCC
Eg-BIP	42	TAAGACATCGGTGCGAGCACTCTGCCTTCGTTAGGTGGAGAT
Eg-LF	25	CCACTGGTAAGTTAAATGCTTTTCC
Eg-LB	24	TTCTCACTCATCTCACTGAATGTG
*Echinococcus multilocularis*		
Em-F3	20	AACCACCAACCTTTCGGTTA
Em-B3	19	GGAATGGGAAGGTGATGGC
Em-FIP	35	GCAGTGTAGCGCGTGGCACAGCCGAACGCGCTAAC
Em-BIP	39	GCAAGCCGCCGCCTCTTCTGATGGTGAGGTAGTGTTGCA
Em-LF	17	AGCCTTCGTGGCGCAAT
Em-LB	20	TCTCTCCCACCACCACCACC

**Table 2:** Positive rate of canine fecal samples detected by LAMP-LFD and ELISA methods.

City	Regions (Number)	Canine Fecal Samples (Number)	LAMP-LFD				ELISA	
			Positive Samples			Positive Rate/%	Positive Samples	Positive Rate/%
			E.m	E.g	Total			
Lhasa City	1	16	3	5	8	50	8	50
Nagqu	8	124	1	4	5	4.03	0	0
Shannan	10	113	1	12	13	11.50	4	3.54
Shigatse	11	189	7	42	49	25.93	52	27.51
Nyingchi	7	146	1	2	3	2.05	0	0
Chamdo	10	238	2	5	7	2.94	0	0
Ngari	7	156	1	11	12	7.69	12	7.69
Total	54	982	16	81	97	9.88	76	7.74

**Table 3:** Positive rate of canine fecal samples detected by ELISA method.

Sample Size			LAMP-LFD		Sensitivity	Specificity
			Positive	Negative		
76	ELISA	positive	73	3	96.05% (73/76)	97.35% (882/906)
906		negative	24	882		

## Data Availability

All the relevant data are provided in the article.
